# Sustainable Valorization of Tomato By-Products to Obtain Bioactive Compounds: Their Potential in Inflammation and Cancer Management

**DOI:** 10.3390/molecules27051701

**Published:** 2022-03-04

**Authors:** Tânia Laranjeira, Ana Costa, Catarina Faria-Silva, Daniela Ribeiro, José Miguel P. Ferreira de Oliveira, Sandra Simões, Andreia Ascenso

**Affiliations:** 1 Faculty of Pharmacy, Universidade de Lisboa, Av. Prof. Gama Pinto, 1649-003 Lisboa, Portugal; tanialaranjeira@campus.ul.pt; 2 Faculty of Pharmacy, Research Institute for Medicines (iMed.ULisboa), Universidade de Lisboa, Av. Prof. Gama Pinto, 1649-003 Lisboa, Portugal; anamartinsmaiacosta@gmail.com (A.C.); anacatarinafs@hotmail.com (C.F.-S.); 3 LAQV, REQUIMTE, Laboratory of Applied Chemistry, Department of Chemical Sciences, Faculty of Pharmacy, University of Porto, 4050-313 Porto, Portugal; daniela.sa.ribeiro@uac.pt (D.R.); jmoliveira@ff.up.pt (J.M.P.F.d.O.); 4 Faculty of Agrarian Sciences and Environment, University of the Azores, 9700-042 Angra do Heroísmo, Portugal

**Keywords:** tomato, tomato processing industry, pomace, peel, seeds, oleoresins, anti-inflammatory, anti-cancer

## Abstract

Tomato producing and processing industries present undoubted potential for industrial discarded products valorization whether due to the overproduction of fresh tomatoes or to the loss during processing. Although tomato by-products are not yet considered a raw material, several studies have suggested innovative and profitable applications. It is often referred to as “tomato pomace” and is quite rich in a variety of bioactive compounds. Lycopene, vitamin C, β-carotene, phenolic compounds, and tocopherol are some of the bioactives herein discussed. Tomato by-products are also rich in minerals. Many of these compounds are powerful antioxidants with anti-inflammatory properties besides modulating the immune system. Several researchers have focused on the possible application of natural ingredients, especially those extracted from foods, and their physiological and pharmacological effects. Herein, the effects of processing and further applications of the bioactive compounds present in tomato by-products were carefully reviewed, especially regarding the anti-inflammatory and anti-cancer effects. The aim of this review was thus to highlight the existing opportunities to create profitable and innovative applications for tomato by-products in health context.

## 1. Introduction

Tomato (*Solanum lycopersicum* L.) belongs to the nightshade family (*Solanaceae*) and is botanically categorized as a fruit. Tomato plants tend to grow preferably at warmer temperatures, and thus their blossoming starts when the weather is hotter and there is more sunlight [1]. First, small yellow/white flowers blossom, and then they eventually fall in order for the fruits to grow. The tomato fruits can vary in color (red, yellow, purple, and green), shape (from oval to spherical), and size (1.5–7.5 cm) [1,2]. From the historical point of view, the first wild species of tomatoes were found in South America, and only later were they domesticated to be used as food [1]. In Europe, the tomato was introduced in the 16th century by the Spanish. Eventually, its usage spread over the globe in a way that now we can contest to its remarkable presence in the most unique cuisines [1].

Tomatoes are rich in lycopene and other carotenoids, flavonoids, vitamin C, K1, B2 and B9, potassium, copper, iron, phosphorous, among many other active compounds [2]. Regarding their mineral and nutritional content, tomatoes have been considered as functional or nutraceutical foods, which means that the regular consumption of this food may prevent and/or act on certain human diseases [2]. Therefore, tomatoes have gained major significance over the past years in human nutrition mainly due to their phytochemical content and potential medical and pharmacological applications [2,3]. Besides tomatoes, the remains from the industry of processed tomato products (including peel and seeds) give rise to other by-products with added value. This review intends to explore the sustainable valorization of tomato processing industry by-products and its usage in the maintenance of human health. Challenges and opportunities will also be addressed in this context. 

The literature search began with the searching of manuscript keywords on PubMed, ScienceDirect, Google Scholar, National Center for Biotechnology Information (NCBI), MEDLINE- National Library of Medicine^®^ (NLM), National Institute of Health (NIH), Food and Drug Administration (FDA), and the Food and Agriculture Organization Corporate Database (FAOSTAT). After an initial screening of titles, a total of one hundred and fifty-six independent studies were identified. The quality and eligibility assessment were performed in full-text documents and a total of twenty-nine studies were excluded because the information was irrelevant.

## 2. Sustainable Valorization of Tomato Processing Industry By-Products

### 2.1. Tomato Industry and By-Products

The tomato industry has been growing worldwide, achieving a total production of 182,256,458 tons in 2018. According to the latest data available, China was responsible for most of the global tomato production, followed by the United States of America (USA) [4,5]. In several countries, tomatoes were the most consumed “vegetable” per capita [6].

Like most large-scale production industries, the tomato industry faces the major issue of the discarding of food and by-products. The fact that tomatoes are especially frail in the presence of external environmental stressors such as high temperature and humidity compounds the problem [7]. On the other hand, the need to ensure that enough fresh tomatoes are available for consumption or even processing into other products leads, in most cases, to overproduction, and, thus, more leftovers. In addition, the overproduction is also a way for the industry to overcome the potential tomato losses involved in the process and the high percentage of tomatoes that do not reach market standards [8].

Food residues have been an issue for most food-producing industries, and it has been challenging to quantify the exact extent of this problem, since there are many stages in the food supply chain in which food can be rejected. Besides being a clear economical problem for the industries involved, it also adds to environmental pollution and to an unbalanced relation between the scale of waste and the malnutrition crisis on a large part of the human population [9].

Although there are individual and organizational initiatives and programs proposed by different governmental institutions, such as EPA (United States Environmental Protection Agency), that continuously try to overcome this problem, food loss is still an unsolved setback in our society [10]. Developed and industrialized countries are nowadays responsible for most of the overproduction and consequent waste [11]. Therefore, it becomes clear that the waste intrinsically involved in producing and processing foods should be not only diminished, but also “recycled” to ensure a sustainable valorization of processing industry by-products.

It was estimated by the EPA that around 31% of all the fresh tomatoes bought by householders were thrown away in the USA. This represents approximately 21 tomatoes per person each year. This loss is estimated to cost over 2.3 billion dollars each year, and it is not exclusively related to the food loss, as it also means the loss of resources such as freshwater, energy, and farming fields [12]. On the other side, around 3–7% of material is lost during tomato processing. This “waste” is commonly called tomato pomace, being mostly made of peels, seeds, and some residual tomato tissue. Despite these by-products being usually discarded, they still are nutrient- and vitamin-enriched sources [8,13].

Additionally, there is another rejected fraction to consider: the unharvest green tomatoes, leaves, and roots. Green tomatoes that are not harvested and remain in the fields are rich in glycoalkaloids such as tomatine. Tomatine comprises two molecules: α-tomatine and dehydrotomatine, which accumulate in every organ of the tomato plant. In the tomato fruit, the content of these two molecules decreases with tomato ripening, being its highest when unripe (green). These fractions usually remain in the fields without further valorization. Nevertheless, many studies described the potential of tomatine for human health. Tomatine has shown to exhibit antioxidant, anti-inflammatory, antibiotic, and anti-fungal properties. Immune-stimulating and cardiovascular effects were also demonstrated. Additionally, tomatine have been demonstrated to inhibit the growth of a large range of cancer cells, such as colon, breast, lung, prostate, among others. Further investigation is necessary since most of the mechanisms of action are still unknown [14,15,16].

### 2.2. Effect of Processing: Quality of the By-Products

Over the past years, the consumption of processed foods has increased mainly due to population growth. Thus, the demand to convert fresh products into useful, profitable, and quality products thrived [17]. Food processing has changed how we eat and live in many ways. Nowadays, we can consume a variety of foods all year round, most of them out of season, allowing a more diverse diet [18]. When it comes to the tomato processing industry, many advantages have been highlighted regarding fresh tomatoes processing (Table 1).

**Figure 1:**
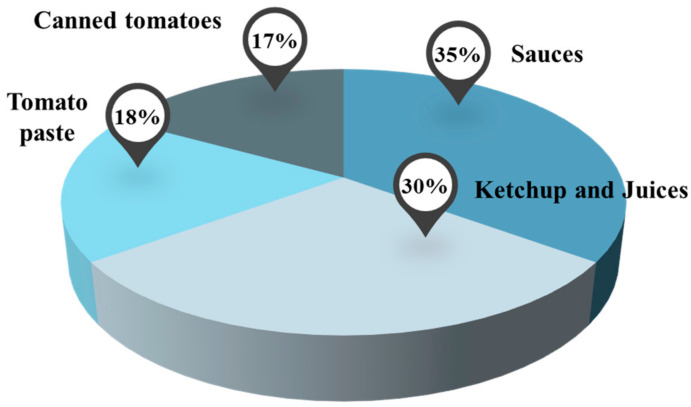
Graphic representation of estimated percentages of tomato processed by-products by USDA [20].

Tomatoes are versatile and can be processed into many different products, such as pastes, juices, soups, jams, sauces, and others [18,19]. To achieve this, the tomato processing industry resorts to different strategies and technologies, taking into account its final purpose [8]. It was estimated by the Economic Research Service of the United States Department of Agriculture (USDA) that 30% of the fresh tomatoes are processed into ketchup and juices, 35% into sauces, about 18% into tomato paste, and 17% into canned tomatoes (Figure 1) [20].

One of the main concerns of all the food processing industries is to ensure the quality of the final processed products, therefore being extremely important to consider which factors affect the raw material. Considering that the quality of the by-products is directly related to the processing technique and conditions employed, it is of utmost importance for the tomato processing industry to be correctly instructed to achieve the best outcomes. The quality of tomato by-products is assessed through appearance, viscosity, taste, acidity, and nutritional content [21]. 

Before its final processing destination, tomatoes are harvested, precisely sorted, soaked, and washed, and, ultimately, trimmed as needed. There are multiple processing strategies, and most of the by-products require a long succession of different procedures to achieve their final form. As an example, tomato paste has to suffer several steps of heating, which include hot-break, drying, and others [8,22]. Heat processing strategies are commonly used, as they allow for destroying microorganisms, enzymes, and eventually, to obtain a more efficient separation between the tomato juice and the pulp. Therefore, the hot-breaking method is one of the most used processes to obtain tomato pulp, juice, soup, and paste. The hot-breaking method consists of rapidly heating the tomatoes to temperatures higher than 77 °C, followed by immediate crushing or chopping. As a result, the obtained tomato pulp presents higher viscosity due to the prevention of pectin (thickening agent) break-down. Pectic enzymes are inactivated by heat, and once these enzymes are destroyed, pectin breakdown is avoided, obtaining a more full-bodied and thicker pulp [22].

Although the hot-breaking method eases tomato homogenization and further handling, some studies have shown that it adversely affects mineral and vitamin content, while in other cases, it has shown to be beneficial [23,24]. Several researchers have found that some bioactive molecules of tomatoes, such as lycopene and β-carotene, may benefit from heat processing and maceration since those techniques can disrupt the tomato cell-matrix, and therefore allow these carotenoids to become more bioavailable [24].

Understanding how processing affects the natural source is extremely important since tomato consumption is encouraged for the prevention of chronic illnesses like diabetes, cardiovascular diseases, and others, due specifically to its mineral and vitamin content. Additional further valorization is also dependent on previous processing [8].

#### 2.2.1. Effect of Processing on Vitamin C Content

Vitamin C or L-ascorbic acid is a powerful antioxidant, well known for its phytochemical properties and importance for human health. The human body is unable to produce Vitamin C, meaning that its presence in the human body relies merely on our diet [25]. Fresh tomatoes are an important source of vitamin C, but processing tomatoes may negatively impact its content [26]. Vitamin C content appears to increase with tomato fruit ripening, from green to red, but once tomatoes are harvest and stored, further maturation and light exposure have been related to vitamin C content decrease [24,27].

**Figure 2:**
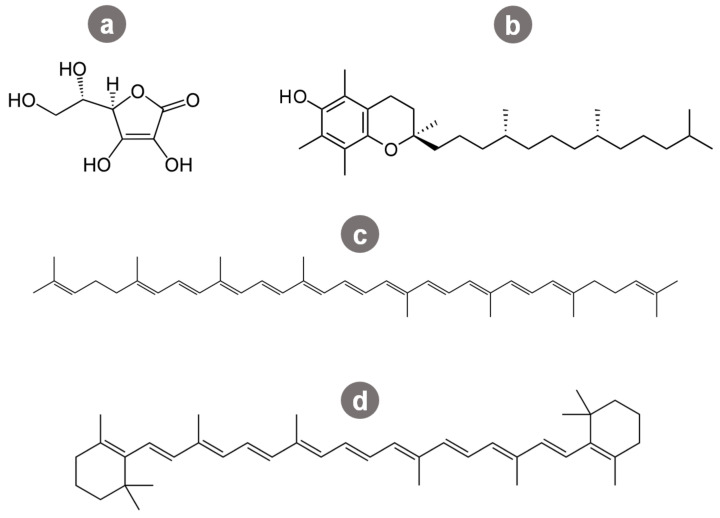
Chemical structure of main bioactives extracted from tomato by-products: (**a**) vitamin C; (**b**) vitamin E; (**c**) lycopene; (**d**) β-carotene.

Vitamin C is a thermolabile and water-soluble compound (Figure 2a), which means that, after applying the most common processing techniques such as grinding, chopping, and heating, its content appears to decrease. During post-harvesting storage, light exposure seems to be the main cause of vitamin C loss since it leads to oxidation. During cooking, vitamin C appears to shed from the tomato matrix into the water, which can be explained by its water solubility [23,24,28].

#### 2.2.2. Effect of Processing on Lycopene Content

On the contrary, lycopene content has been shown to increase after certain processing techniques such as heat processing [23,28]. Lycopene is a carotenoid responsible for giving tomatoes their red pigmentation. It possesses 2 non-conjugated and 11 conjugated double-bonds (Figure 2c), and it is due to this arrangement of the conjugated double bonds that lycopene is more susceptible to degradation via enzymes, oxidation, isomerization, and others.

The main pathways of degradation of lycopene during tomato processing are oxidation and isomerization. Oxidation occurs preferably at a low pH, in the presence of light and oxygen during non-thermal processing, like cutting, grinding, and even during the storage period. Thermal processing, on the other hand, allows tomato tissue breakdown, meaning that most of the bonds become disrupted [29]. Additionally, it seems that heat processing may favor isomerization. Although most identified compounds are naturally all trans-forms, cis-isomers present higher bioavailability since they are more easily absorbed in the intestine. The cis-isomers also possess improved antioxidant capacity, and, thereby, heat processing and consequent isomerization are desirable [23,29,30]. 

#### 2.2.3. Effect of Processing on β-Carotene, Phenolic Compounds, and Vitamin E Content

β-Carotene is another carotenoid, responsible for giving fruits and vegetables its characteristic orange color, being the second most abundant colored carotenoid in tomatoes (Figure 2d). β-Carotene is widely used in the food industry as an additive, specifically as a food coloring agent. Its main significance in human health is related to its antioxidant capacity and to be a precursor of vitamin A. Some studies have shown that short-term heating may decrease or, on the contrary, not affect the β-carotene content in processed tomato by-products (e.g., soups and sauces). Other research showed that this content may become more available. Therefore, additional studies are still required in this matter [24,31,32].

Phenolic compounds are important phytochemicals with powerful antioxidant activity present in most fruits and vegetables. These compounds can be divided into two main categories: polyphenols and phenolic acids. Their antioxidant capacity is primarily related to reactive species’ scavenger properties and electronic and atomic exchanges. In tomatoes, some of the phenolic compounds are flavonoids, phenolic acids, and tannins. Phenolic content in tomatoes is significantly conditioned by farming techniques, genotype, and storage. In most fruits and vegetables, phenolic compounds have been found mostly bonded to cell walls. Therefore, some processing strategies, especially those that cause membrane rupture, increase the bioavailability of phenolic compounds. Similar to what happens with lycopene after heat treatment and homogenization, it appears that these transformations also impact the phenolic content in tomatoes in a positive manner [24,33].

Vitamin E is a powerful liposoluble antioxidant that comprises eight molecules: α-, β-, γ-, and δ-tocotrienol and α-, β-, γ-, and δ-tocopherol. α-Tocopherol is a compound with the major significance for human health (Figure 2b). Tocopherols, protective antioxidants in plants, are one of the most abundant vitamins in tomatoes. Vitamin E content in tomatoes is deeply dependent on environmental conditions such as heat and light. When discussing how processing affects the vitamin E content in tomatoes, different results have been found. Some studies have shown that α-tocopherol content is not affected by processing since they found the same amounts in fresh tomatoes and processed tomato paste. On the other hand, γ-tocopherol has been found at a lower percentage in the final product. The same researchers reported that γ-tocopherol was mainly present in seeds and peels, while α-tocopherol was present in all parts. This explains the lower levels of γ-tocopherol in the tomato paste since seeds and peel are discarded in this production process. Regarding the tomato juice production, it appears that sterilization and homogenization, decreases α-tocopherol. Short-term heat processing seems to be beneficial and to increase some tocopherols’ content, while long-term heat processing results in their degradation [20,24,34].

Since processing affects the content of the various bioactive compounds differently, it is important to consume fresh tomatoes to overcome any nutritional deficiencies that may occur. This is of extreme importance for thermolabile compounds such as vitamin C, which decrease after processing.

### 2.3. Added-Value of Tomato By-Products

In addition to producing marketable products directly from fresh foods, industries have been equally focused on the research and development of innovative applications of tomato industrial by-products [13]. It is essential to distinguish the biomass that results from the overproduction of fresh tomatoes from the biomass obtained from tomato processing. Both biomasses are different, and, therefore, further applications will differ as well. The biomass that results from overproduction is mainly derived from tomatoes that do not reach market standards, whether due to its color, shape, or size. Over the years, industries have tried to develop technologies to add value to these fractions. The main challenge has been related to its high water content and fast rotting [8]. On the other hand, waste fractions that result from tomato processing are normally depreciated and used either to feed animals, fertilize soil, or are disposed as waste. The biomass that results from tomato processing (“tomato pomace”) is mainly made of seeds, peel and some residual vascular tomato tissue [13].

It is important to note that the tomato fractions disposed of in the environment and the unharvested tomatoes that remain in the fields can lead to an environmental hazard. These fractions, once discarded in the environment, are further disposed of by anaerobic fermentation. As a consequence of this biochemical process, biogas composed of methane (CH_4_), carbon dioxide (CO_2_), nitrogen (N), and others is produced. These are considered greenhouse gases with well-known environmental side effects but also gases with possible further applications. In recent years, biomass technology specifically using waste fractions to produce fuel has gained much interest. Besides being an alternative to the usage of fossil energies, this could further valorize the rejected fraction, not only from tomatoes but also from other foodstuffs. Since CH_4_ emission from tomato decomposition is a problem, alternatively use these fractions for the production of fuel emerges as an interesting alternative, not only beneficial for the environment but also for the industries and chains that produce such excess, allowing further valorization [35,36].

#### Tomato Pomace (Peel and Seeds) and Oleoresins

Tomato pomace is a source of protein, fat, and dietary fibers. Dry tomato pomace usually contains around 12% fat, 20% protein, and 30% raw fiber [37,38]. Approximately 60% of the total tomato pomace corresponds to seed fractions. Tomato seeds have shown to be an interesting source of fat, such as palmitic and oleic acids, and protein, containing higher quantities of lysine and threonine. Tomato seeds have also been shown to be a potential source of trace elements such as Sodium (Na), Calcium (Ca), Magnesium (Mg), Potassium (K) and others. The peel is a source of other amino acids (valine, lysine, and leucine) and trace elements (Mg, Na, K, and Ca). Additionally, tomato pomace has shown to be a source of Manganese (Mn), Iron (Fe), Copper (Cu), Zinc (Zn), naringenin and chlorogenic acid [8,39]. Tomato pomace can also be a good source of dietary fiber (DF), and several studies suggest that tomato seeds and peel have higher contents of polyphenolic compounds [37,40,41].

Tomato pomace, and, more specifically, wasted tomato peel fractions, are also valuable materials for the extraction of carotenoid-rich oleoresins (extraction techniques detailed in Supplementary Material, and in Figure S1 and Table S1). The importance of these extracts for the pharmaceutical and food industries will be described below.

Oleoresins are a combination of pigments, fatty acids, fats, sterols, flavor compounds, and others, that are usually extracted by successive conventional solvent extraction methods. Oleoresins usually contain concentrates of the aroma, active fractions, and flavor of their source. Tomato oleoresins are semi-solid mixtures with a lipophilic nature constituted by essential oil and resins. Tomato oleoresins are promptly absorbed in the organism and act as powerful antioxidants. Besides leading to in vivo health benefits, these oleoresins can also be incorporated in supplements and foodstuffs, for numerous reasons, namely, to improve nutritional content, color, flavor, and other chemical properties. Lycopene-oleoresin is one of the most requested, not only by the food industry, but also by the cosmetic and pharmaceutical manufacturing industry [42,43,44,45].

Tomato oleoresins are commonly extracted by conventional organic solvent or supercritical CO_2_ extractions. These extraction techniques can be applied on their own or combined with other methods. When combined with enzyme extraction methods, that use pectinolytic, cellulolytic, or any others that allow cell wall destruction, higher oleoresin extraction yields can be achieved since the active fraction is more bio-accessible after cell wall rupture [46].

Once an effective extraction of the tomato oleoresins is obtained, further application and manipulation can be diversified. Currently, tomato oleoresins are mainly used by the food industry. Their powerful antioxidant capacity and pigmentation make these oleoresins excellent natural food additives. In the meat industry, tomato oleoresins are commonly used as a natural colorant or antioxidant. Adding tomato oleoresins to meat products allows enhanced shelf life, increased meat redness and further delaying of meat discoloration, reduced lipid oxidation, and off-flavor formation, during the storage period and refrigeration. Hence, tomato oleoresins can work as a natural additive to maintain the integrity and quality of meat products [47,48]. In addition, lycopene-rich oleoresin extracted from tomato by-products, such as wasted tomato peels, acts as an effective dietary stabilizer for some refined oils. Lycopene oleoresin functions similarly to other commonly added preservatives against oil oxidation, which naturally occurs during the storage period. In fact, this oleoresin can be used as a natural preservative, stabilizer, and pharmaceutical active compound [49]. Regarding the in vivo health benefits of oral supplementation of tomato oleoresin, interesting results have been found. The cholesterol-lowering effects observed with lycopene supplementing have also been observed with tomato oleoresin supplementation. These findings suggest that tomato oleoresin has an even higher antiatherogenic capacity than lycopene alone. Tomato oleoresin supplementation, specifically lycopene-rich oleoresins, have been demonstrated to modulate and prevent some diseases. Accordingly, DNA and protein protection and lower tumor burden have been observed after supplementation with this oleoresin [50].

## 3. Biological Properties of Tomato Bioactives

As mentioned above, tomato by-products are a source of minerals, such as Sodium (Na), Calcium (Ca), Magnesium (Mg), and Potassium (K), and are all electrically charged. Electrolytes are essential for human health, as they maintain the body’s homeostasis, i.e., blood volume, fluids quantity, pH levels, and proper muscle, nerve, heart, and brain function [51]. Nevertheless, the consumption of these electrolytes needs to be adjusted. Overconsumption of sodium leads to high blood pressure, while not consuming enough potassium also contributes to this problem. This imbalance plays a major role in the burden of cardiovascular diseases [52]. Therefore, the American Heart Association recommends a daily sodium intake not higher than 1500 mg for most people. It is important to note that the overconsumption of sodium is mostly related to the ingestion of processed foods and meals prepared in restaurant chains. While fruits, vegetables, and meats are natural sources of sodium and other micro-nutrients, their consumption does not seem to be associated with higher sodium blood levels [53].

Some studies have found that increased consumption of potassium can be an effective way to treat and prevent hypertension, and, therefore, decrease the risk of cardiovascular disease [52]. A healthy intake of potassium is encouraged not only for its vascular benefits, but also for reducing water retention and bone mass loss, protecting against kidney stones, strokes, and osteoporosis, respectively [52,54,55]. Cardiovascular diseases, specifically strokes and ischemic heart disease, are the main cause of death worldwide according to WHO (World Health Organization) [56]. 

Magnesium acts as a cofactor for many enzymes, and a regulator of different essential functions, such as glycemia, blood pressure, muscle contraction, and neuromuscular transmission. Magnesium equally participates in energy and nuclear materials production, in active transmembrane transport and bone development [57].

Calcium is one of the most abundant minerals in the human body, being mostly distributed in the teeth and bones. Only a small part of the total calcium (around 1%) corresponds to serum calcium. This mineral is vital for bone development and health, muscle contraction, intracellular signaling, heartbeat control, blood clotting, hormonal secretion, and nerve transmission [58,59,60].

Oleic acid, also present in tomato seeds, is a monounsaturated fatty acid. The replacement of saturated fats with oleic acid or other monounsaturated fats appears to be beneficial for human health, including the maintenance of healthy cholesterol levels, modulation of insulin sensitivity, blood pressure, and inflammatory markers, among others [61].

Palmitic acid, although being described as an unhealthy saturated fat, contributes to different physiological activities. It is one of the most plentiful saturated fatty acids in the human body. It is present in membrane phospholipids and adipose triacylglycerols. This fatty acid acts as a surfactant in the lungs and is also essential for maintaining physical properties of membranes. Over the years, some studies have established a relationship between breast cancer and palmitic acid blood levels. Although some studies stated the opposite, palmitic acid health benefits are still controversial [62].

Dietary fiber (DF) is usually defined as plant-derived polysaccharides that are indigestible and non-absorbed by the human gastrointestinal tract. Despite not having an actual nutritional value, DF is indispensable for human health. DF seems to act on the prevention of gastrointestinal related disorders and heart diseases. Soluble fibers appear to improve levels of LDL cholesterol, while insoluble fractions may increase bowel movements and feed healthy bacteria, acting as probiotics. Moreover, DF can bond with heavy metals, aiding the natural body detoxification process [37,41]. 

The effects of oral supplementation and/or topical application of tomato-containing bioactives are discussed in Supplementary Materials.

### 3.1. Anti-Inflammatory and Anti-Cancer Activity of Lycopene

**Figure 3:**
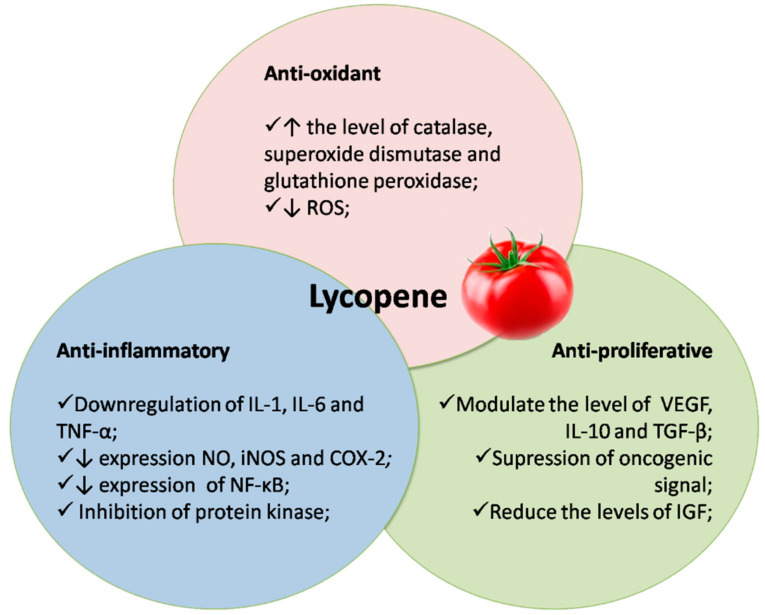
Schematic representation of the main mode of action of lycopene as anti-inflammatory and anti-cancer agent.

Lycopene is one of the most studied natural compounds present in tomato and tomato by-products due to its anti-inflammatory and anti-cancer properties. The link between inflammation and cancer development and progression has been a topic of study in the last years. During chronic inflammation, the persistent and unresolved inflammatory process forms a harsh environment that favors immunosuppression, tumorigenesis, and metastasis formation. The main cells responsible for cytokine production at the inflammation sites are macrophages and T lymphocytes that will be responsible for the persistent and unresolved inflammatory response, and the production of reactive species than can damage the DNA, nearby cells, and tissues (Figure 3) [63,64].

#### 3.1.1. Anti-Inflammatory Activity 

Lycopene generally exerts its anti-inflammatory activity through the following mechanisms: the downregulation of proinflammatory cytokines, such as IL-1, IL-6, TNF-α [63,65], and the reduction of other proinflammatory mediators, such as NO production, iNOS and COX-2 expression, and PEG2 production [63,65,66]. Another anti-inflammatory strategy is the reduction of oxidative stress that suppresses the expression of nuclear factor-kappa B (NF-κB), inhibiting in this way the expression of proinflammatory cytokines [67,68]. Another pathway for inflammation reduction is through the inhibition of ERK/p38 MAP kinase in macrophages [69]. Most studies evaluate the influence of lycopene in the inflammation regulation using cell lines or in vivo animals, but its effects in clinical trials are also described. Table 2 summarizes different clinical studies that explored the effect of tomato-based products or lycopene supplementation in reducing inflammation. 

Stahl et al. [70] designed a study to investigate whether intervention with a natural dietary source rich in lycopene may protect against UV-induced erythema in humans. For that purpose, tomato paste (providing 16 mg/d of lycopene) was ingested with olive oil. By 10 weeks of tomato paste consumption, lycopene levels increased in the serum of the subjects and the erythema formation, an indicator of the sunburn reaction, was 40% lower. Interestingly, in 2003, Heinrich et al. [71] also demonstrated this erythema-protective effect, but through the administration of a mixed carotenoids soft gel capsule (β-carotene, lycopene, and lutein). This protection against cutaneous photodamage in humans by the ingestion of a tomato paste rich in lycopene was also reported by Rizwan et al. [72]. They observed protection against acute and potentially longer-term aspects of photodamage, by the inhibition of ultraviolet (UV)R-induced MMP-1 expression in the dermis, the reduction of the collagenolytic activity, and the increase of collagen synthesis. Also observed was a potential regenerative response to the UVR insult facilitated by the antioxidant lycopene. Finally, following supplementation, the UVR-induced reduction was abolished in the groups treated with the tomato paste. More recently, a lycopene-rich tomato nutrient complex was demonstrated to be able to protect against UVA/B and UVA1 radiation at a molecular level. This complex fully prevented UVA1- and UVA/B-induced upregulation of matrix metallopeptidase 1 mRNA, heme-oxygenase 1, and intercellular adhesion molecule 1. 

In what concerns the amelioration of inflammatory-related chronic diseases, lycopene has also been demonstrated to be a potential pharmacologic alternative. Upritchard et al. [73] conducted a clinical trial where tomato juice, vitamin E, and vitamin C were administered to type 2 diabetes patients. The authors observed a nearly 3-fold increase of the plasma lycopene levels, during supplementation with tomato juice, which was associated to an intrinsic resistance of LDL to oxidation. This effect was almost as effective as the supplementation with vitamin E.

Oral lichen planus (OLP) is a common chronic inflammatory mucocutaneous disease of the oral mucosa with unknown etiopathogenesis. Lycopene (from natural tomato extract), when administered through soft gel capsules to patients with erosive OLP recalcitrant to topical steroids, were demonstrated to decrease serum 8-isoprostane levels. This effect manifested as reduction of the size of oral red lesions, regression of their severity, and conversion of erosions and ulcers to erythema or to disappearance [74]. Kushwaha et al. [75] also conducted a clinical trial where they report lycopene capsules effective on the management and prevention of OLP lesions through the complete or partial remission of lesions. In a 30-day clinical trial in patients with coronary vascular disease, patients were supplemented with lycopene ingested either in the form of lactolycopene or in the form of lycosome-formulated GA. Lycosome formulation led to the reduction in Chlamydia pneumoniae IgG and in the inflammatory oxidative damage marker. The decrease in oxidized LDL caused by lactolycopene did not cause any significant effects. Therefore, enhanced bioavailability of lycopene promotes its antioxidant and anti-inflammatory effects, and favors a positive effect on the cardiovascular system [76].

However, in a double blind, randomized, placebo-controlled crossover trial, in participants who had ingested a supplement containing a tomato extract (lycopene, phytoene, and phytofluene), no effect was observed on inflammation and oxidative stress despite the observed attenuation of the post-exercise increase in the muscle damage biomarker myoglobin [77]. Indeed, a recent systematic review [78] evaluated the effect of lycopene on the inflammation and the consumption of lycopene supplements and tomato products on the biomarkers of inflammation in humans. It was indicated that the lycopene levels were negatively affected during the inflammation. Although an increase of lycopene levels (serum or plasma) was observed after intake of tomato /lycopene supplementation, there was no change in the inflammatory biomarkers. The authors of this study pointed that there is little evidence that the increase of consumption of tomato or lycopene supplements reduces the inflammation, but the depletion of lycopene could be one of the first indicators of low-grade inflammation [78]. 

#### 3.1.2. Anti-Cancer Activity—Inhibition of Skin Cancer

Inflammation is closely associated with cancer development. When acute inflammation does not resolve, chronic inflammation may occur, originating a suitable microenvironment (with macrophages, lymphocytes, proinflammatory cytokines, reactive oxygen species (ROS), signaling molecules) that may activate oncogenes, damage the proteins and DNA, playing in this way a role in cancer development, invasion, and metastasis formation [64]. Due to the anti-inflammatory properties of lycopene, this natural compound has been explored as an anti-cancer agent, and there are many studies that evaluated the effect of lycopene on tumorigenesis using different cell lines and in vivo models. Studies indicated that lycopene could increase the levels of antioxidants defenses, particularly catalase, superoxide dismutase or even glutathione peroxidase, enabling the reduction of ROS [80,81]. This compound also modulated the levels of proinflammatory cytokines [tumor necrosis factor-α (TNF-α), IL1, IL6 and IL8] [82], the cytokines involved in immune evasion [transforming growth factor-beta (TGF-β) and IL10] [82] and the angiogenesis process (VEGF)] [83,84]. The proinflammatory signaling also diminished after treatment with lycopene by reducing the levels of phosphorylation of NF-κB p65, STAT3 and IL-6 protein and through the suppression of some oncogenic signals, (mTOR complex 1 activation, Met mRNA, β-catenin protein) [85]. Moreover, in vivo studies indicated that lycopene could decrease the tumor growth in different xenograft models [82,86,87].

According to GLOBOCAN estimates in 2020, colorectal cancer is the second leading cause of cancer-related mortality, with more than 935,000 deaths occurring annually [88]. Oral administration of lycopene is expected to result in the highest levels of lycopene delivered to the gastrointestinal tract, and studies with human subjects revealed that administration of tomato-derived lycopene was associated with lower insulin growth factor (IGF)1 plasma levels, a growth factor responsible for the cancer cell growth [89]. In addition, lycopene from tomato increased the serum levels of IGF binding protein (IGFBP) 1 and 2, which decreased the binding of IGF-1 and 2 to the corresponding receptors, thereby preventing cell growth activation [90]. In an additional study in colorectal cancer patients, lycopene was associated with skin protection against cancer treatments [91].

Prostate cancer is the second leading cancer in males [92]. In two studies, lycopene administration significantly decreased the levels of prostate-specific antigen (PSA) in prostate cancer patients [93,94]. Moreover, in leukocytes of prostate cancer patients, lycopene decreased the levels of 8-hydroxy-2′-deoxyguanosine (8-OHdG), a marker of DNA damage [95].

In women, breast cancer is the second leading cancer [92]. For a population with a high-risk of breast cancer, lycopene decreased the level of free IGF-1 [96]. In a study with breast cancer patients, lycopene prevented some of the negative effects of therapy to the skin, and lycopene levels were inversely correlated with urinary 8-OHdG [79].

Skin cancer incidence, particularly melanoma, has been increasing worldwide. Globally, approximately 230,000 reports of malignant cancers diagnosed correspond to melanoma cases, which cause about 55,000 deaths annually, and has increased more dramatically than any other malignancy [97]. Indeed, it is projected that the number of newly diagnosed cases will double by 2030 and that the annual cost of treatment will triple [98]. In a retrospective study with human subjects, higher intakes of lycopene from several food products were found to decrease the risk of squamous cell carcinoma [99].

The above-mentioned clinical studies linking lycopene consumption to anti-cancer effects are summarized in Table 3. 

## 4. Challenges and Opportunities

**Figure 4:**
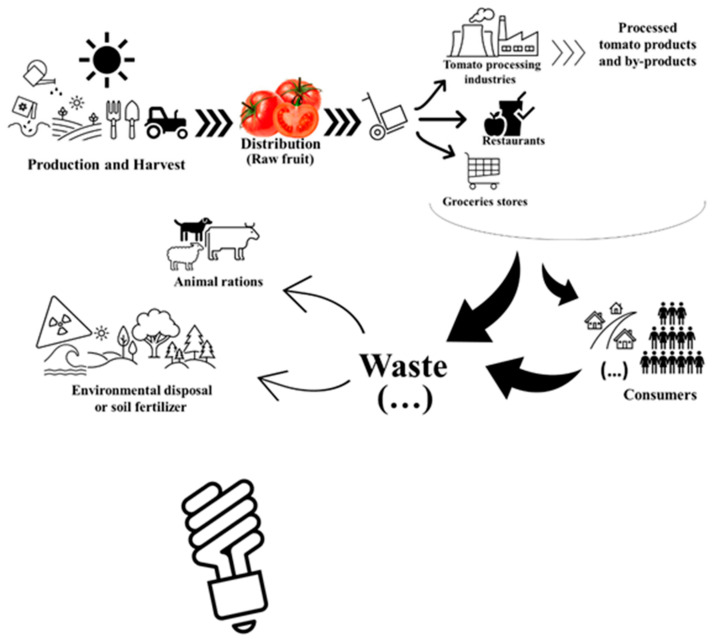

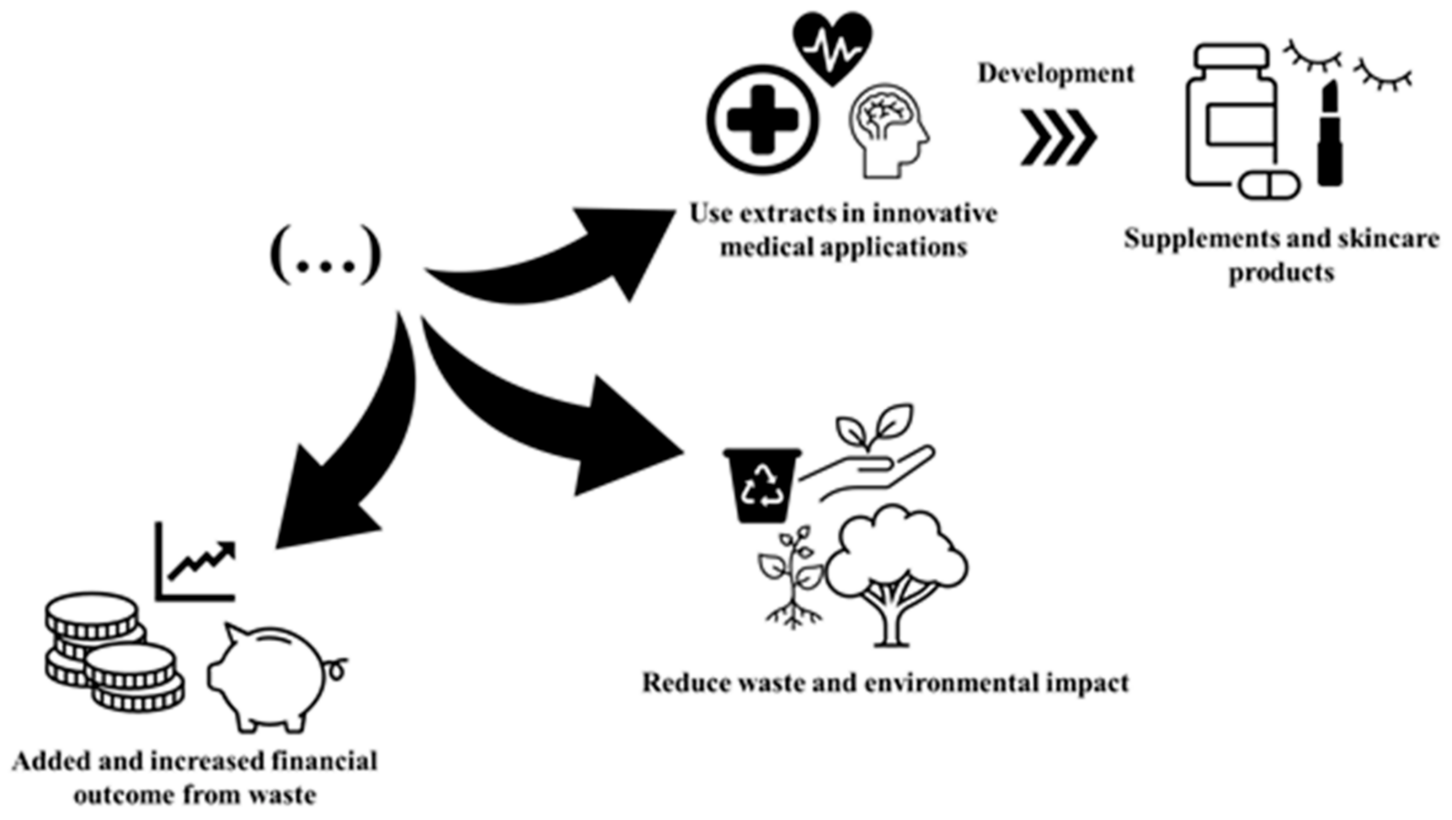
Schematic representation of traditional tomato production and processing circuit versus added-value circuit.

The tomato industry is a global and large-scale manufacturing industry with considerable product loss. Potential added-value by-products could be thus explored for alternative applications (Figure 4). 

The traditional tomato production and processing pathway discard rotten or non-standard “fresh-tomatoes” and by-products either as environmental waste, or as non-valued materials used in the manufacture of soil fertilizer or animal rations. The opportunity to reduce such by-products and eventually find additional processing methods could be quite interesting for most tomato processing industries. As the tomato industry represents one of the major vegetable production industries over the globe, the results of the trial and implementation of new procedures or by-products applications could be readily accessed. This large-scale dimension allows a more flexible approach since fresh tomatoes (that do not reach market standards) or by-products are not yet considered as “raw-materials”. Therefore, they would not face ethical issues in contrast to perfectly eatable fresh tomato fruits for the research of novel applications. 

One of the main opportunities for the tomato processing industries is related to the increased industrial income and possible medical discoveries whether tomato pomace becomes an added-value material, or the extraction and isolation processes allow for obtaining target tomato bioactive compounds. The supplementation of tomato bioactive extracts (including vitamins and minerals) has shown positive outcomes for human health in modulation and treatment of certain diseases. Nevertheless, more studies should be focused on the long-term effects of tomato bioactive on the human body. 

Additionally, the opportunity to transform these industries into more environmentally friendly ones emerges. The initial “waste” can be reused to extract bioactive compounds with “eco-friendly” methods. 

While this matter appears as a potential income of these industries, there are still some challenges to consider, such as: (a) available and strict studies demonstrating efficient and profitable applications of tomato by-products; (b) defined standard procedures or techniques for optimal extraction of bioactive compounds and moisture of fresh raw material; (c) materials, equipment, and trained professionals still needed for this new purpose. To achieve an efficient and profitable use of the industrial leftovers, the companies require a paradigm shift, i.e., stop looking at tomato by-products as waste and see it as added-value material.

In conclusion, tomato by-products no longer need to be discarded. As extensively discussed in this review, there are plenty of interesting and sustainable applications for tomato by-products. The fact that tomato by-products contain high levels of anti-inflammatory and antioxidant compounds (specially lycopene), with the ability to modulate the development of certain disorders such as cancer, shows a potential to be further explored in this context. Additionally, since tomato by-products are not yet considered as valued raw materials, the possibility for the investigation and development of further applications is facilitated and could be conducted at very low cost, with significant profitable outcomes. It is important to note that there are still not many studies available that prove the efficiency of these innovative applications. Pioneer industries would require multidisciplinary teams to efficiently develop such products and analyze the stability of these compounds when incorporated in different formulations.

## Figures and Tables

**Table 1:** Advantages and Disadvantages of tomatoes processing [18].

Advantages	Disadvantages
Extended food lifespan and storage period	Not fresh and with preservatives
Consumption out of season and a more diversified diet	More caloric than fresh tomatoes
Facilitated food storage	Decreased specific bioactive content (e.g., thermolabile molecules as vitamins) unlike others (e.g., *cis*-lycopene)
Higher stability	More expensive to consumers
Decreased tomato losses	
Increased market value of tomatoes	

**Table 2:** Clinical studies to assess the anti-inflammatory properties of lycopene.

Supplements/Diet	Dose	Study	Outcomes/Observed Effect	Reference
Lycopene in soft gel capsules	10 mg lycopene/day, 8 weeks	Patients with erosive oral lichen planus recalcitrant to topical steroids	Higher reduction of the 8-isoprostane levels (biomarker of lipid peroxidation and oxidative stress) in the group treated with lycopene	[74]
Lycopene supplements	4 mg lycopene/day, 8 weeks	13 patients with symptomatic oral lichen planus	11 patients showed a partial remission of the lesion; a complete remission was observed in 2 patients.	[75]
Tomato paste	16 mg lycopene/day, 10 weeks	22 participants, type II skin, UV light at dorsal skin	Significant reduction of erythema.	[70]
Lycopene softgel capsule	8 mg lycopene/day, 12 weeks	24 participants, type II skin, UV light at dorsal skin	Significant reduction of erythema.	[71]
Tomato paste	16 mg lycopene/day, 12 weeks	20 participants, type I or II skin, UV light at upper buttock skin	Significant increase in the minimal UV dose required to cause erythema; inhibition of MMP-1 expression induced by UV.	[72]
Lycopene softgel capsule	20 mg lycopene/day, 12 weeks	33 participants, UV light at upper buttock skin	Inhibition of HO-1, MMP-1 and ICAM-1 expression induced by UV.	[79]
Supplements of tomato extract	11 mg lycopene, phytoene, and phytofluene, 4 weeks	20 participants (endurance runners)	No significant differences were observed in the oxidative stress and inflammation after exercise.	[77]
Lycopene supplements (form of lactolycopene and lycosome-formulated GA lycopene)	7 mg lycopene/day, 30 days	69 patients with coronary vascular disease supplemented with a daily dose of lycopene in the form of lactolycopene;74 patients supplemented with the form of lycosome-formulated GA lycopene	Reduction of *Chlamydia pneumoniae* IgG and the markers related with oxidation (inflammatory oxidative damage, oxidized LDL) in the lycosome-formulated GA lycopene.	[76]
Tomato juice	tomato juice (500 mL/day), 4 weeks	57 patients with type 2 diabetes	Consumption of tomato juice increased the resistance of LDL to oxidation.	[73]

**Table 3:** Clinical studies to assess the anti-cancer properties of lycopene.

Supplements/Diet	Dose	Study	Outcomes/Observed Effect	Reference
Tomato lycopene extract	30 mg lycopene/day, 10 ± 2 days	Colon cancer patients	Reduction in 25% of insulin-like growth factor (IGF)-1 in plasma of the patients.	[89]
Tomato-derived lycopene	30 mg lycopene/day, 8 weeks	40 men and 31 postmenopausal women with: a personal history of colorectal adenoma; a family history of colorectal cancer; and both situations	The concentration of IGFBP-1 increased in women after lycopene supplementation;The level of IGFBP-2 at serum increased in women and men after lycopene supplementation.	[90]
Lycopene from tomato oleoresin embedded in whey protein matrix	20 mg/day, duration of the treatment	28 patients with metastatic colorectal cancer (13 also treated with lycopene)	Reduction of skin toxicity induced by treatment; decrease of the mean malondialdehyde index	[91]
Lycopene supplementation	15 mg lycopene twice daily, 3 weeks	Phase II clinical trial:26 men with newly diagnosed prostate cancer	The levels of plasma PSA decreased by 18% in the group treated with lycopene supplementation, but increased in 14% for the control group; the expression of Cx43 was 0.63 and 0.25 in supplementation and control group, respectively.	[93]
Lycopene as tomato sauce-based pasta dishes	30 mg lycopene/day, 3 weeks	60 men with adenocarcinoma of the prostate	After the consumption of tomato sauce, the level of PSA and leukocyte DNA 8-OH-deoxyguanosine/deoxyguanosine (marker of DNA damage) decreased in 17.5% and 21.3% in comparison with control group, respectively; the apoptotic index increased in the hyperplastic and neoplastic of the resected tissue	[95]
Tomato products and tomato products combined with other nutritional substances (selenium, omega-3 fatty acids, soy isoflavones, grape/pomegranate juice, and green/black tea)	30 mg lycopene/day, 3 weeks	79 patients with prostate cancer	Median PSA decreased in 2.9% in the tomato group as compared with control diet; the largest reduction in PSA was observed in patients who had the highest increase of lycopene, selenium and C20:5 *n*-3 fatty acid in the plasma.	[94]
Tomato-derived lycopene supplementation	30 mg lycopene/day, 2 months	Premenopausal women: 24 with history of breast cancer and 36 with family history of breast cancer	Lycopene supplementation did not change the level of total serum IGF-1 in both populations of the study; in breast cancer survivors, the levels of IGF-1 and IGFBP-3 increased; for the population with high-risk of breast cancer, the levels of free IGF-1 decreased after supplementation.	[96]
Tomato juice	160 g juice/day, 6 months	23 patients with breast cancer subjected to radiotherapy	Serum lycopene concentrations significantly increased from end of radiotherapy to final period of consumption of tomato juice (~0.3 to 0.8 µmol/L); skin moisture increased; no change in urinary 8-hydroxy-2′-deoxyguanosine (8-OHdG) levels was observed; however, there was negative correlation between lycopene and 8-OHdG levels.	[79]
Various food products (questionnaire, epidemiological study)	variable	123,570 participants, 3978 cases of squamous cell carcinoma.	Higher intakes of lycopene were significantly associated with decreased risk of squamous cell carcinoma.	[99]

Insulin-like growth factor (IGF-1); Insulin-like growth factor binding proteins (IGFBPs).

## Supplementary Materials

The following supporting information can be downloaded online, Figure S1: Common bioactive compound extraction techniques used in the agriculture industry (adapted from [100]); Table S1: Summary of advantages, disadvantages and extracted compounds in tomatoes by different methods. Refs. [101,102,103,104,105,106,107,108,109,110,111,112,113,114,115,116,117,118,119,120,121,122,123,124,125,126,127] are cited in the Supplementary Material text.
