# Supplementary material for: Sustainable Valorization of Tomato By-Products to Obtain Bioactive Compounds: Their Potential in Inflammation and Cancer Management
Source: Molecules. 2022 Mar 4;27(5):1701. doi: 10.3390/molecules27051701 (PMC8911995; doi:10.3390/molecules27051701)
Supplement: Supplementary file 1 [file molecules-27-01701-s001.zip › molecules-1605706-supplementary.pdf]

molecules

1. Extraction of Tomato By-Products

As most tomato by-products are still used as waste or in animal rations [13], industries are not targeting the nutritional content of tomato by-products yet. In fact, this should be taken into consideration for extracting bioactive compounds during tomato processing or from fresh tomatoes.

Presently, the most common used and traditional extraction techniques involve leaching processes and usage of organic solvents which may be environmental hazard and toxic besides affecting the content of bioactive molecules, thus decreasing its added value. Therefore, it has been made an effort to replace these techniques by innovative and environment friendly ones (Figure S1) [100].

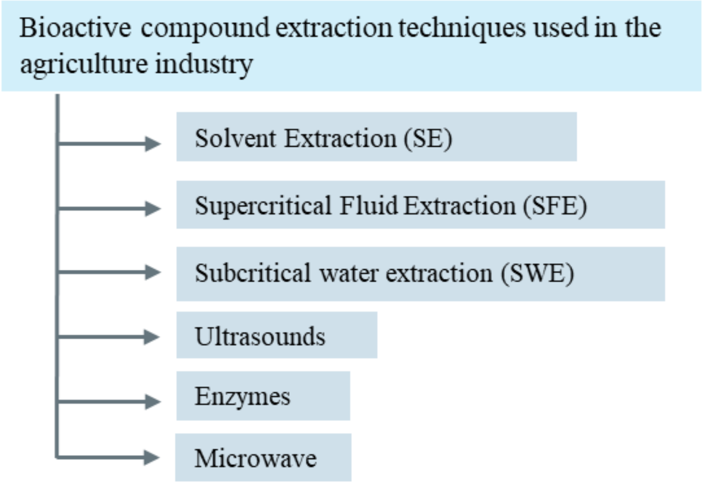

Figure S1. Common bioactive compound extraction techniques used in the agriculture industry (adapted from [100]).

1.1. Solvent Extraction (SE)

The Solvent Extraction (SE) method consists of exposing suitable raw material to organic solvents. Different organic solvents will allow the separation and extraction of the soluble compounds of interest and some natural pigments like anthocyanins. In the SE method, samples are subjected to centrifugation and filtration to remove solid residue and isolate the extracts to be used in the manufacture of food supplements, food additives, or functional foods. The SE method is efficient to purify carotenoids, such as β-carotene and lycopene. Some studies have shown a recovery rate of carotenoids, between 50-96%, when using the SE method. To accomplish a maximum recovery, the proper selection of solvent systems, the temperature, and time (duration of the process), is quite necessary. Some of the benefits of this technique are low processing costs and simplicity to perform it. However, the main concern is the use of high amounts of toxic solvents. Additionally, since solvents reach high temperatures during the extraction, degradation of bioactive compounds might also occur. Therefore, this method has been improved or even replaced by others discussed further ahead [100, 101].

1.2. Supercritical Fluid Extraction (SFE)

The Supercritical Fluid Extraction (SFE) is a solvent-free, and thus, non-explosive, non-toxic and environment-friendly method. The selection of the supercritical fluid is of utmost importance since it will determine the suitable performance of the process. The principle of this method relies on the direct correlation between solvent power and density. To perform the SFE method, the raw material is introduced into an extraction container at controlled temperature and pressure with the supercritical fluid which allows

the dissolution and separation of the compounds from raw material. After separation, both dissolved compounds and fluid are collected. The fluid can be easily removed from the final product. Since the critical temperature of CO2 is close to the ambient temperature, SC-CO2 method is able to extract thermolabile substances [100, 102]. The SFE allows the extraction of different bioactive (including lipophilic) compounds, such as catechins, flavonoids, carotenoids, tocopherols, among others.

1.3. *Supercritical Water Extraction (SWE)*

The Supercritical Water extraction (SWE) is an emerging alternative for the extraction of phenolic compounds from food and food wastes. The term supercritical water refers to water in the liquid state at 100 ºC to 374 ºC and below the critical pressure (22 MPa). This method is also an environmentally friendly technology, and it appears to be one of the most promising approaches to extract multiple bioactive compounds from plants while maintaining its reduced impact on the environment. Some other advantages of SWE are higher quality as well as short time extractions, and low solvent costs [100]. SWE is not commonly used in tomatoes, nevertheless it might be promising for the extraction of flavonoids and tannins.

1.4. *Ultrasounds: Ultrasound-assisted extraction (UAE)*

The Ultrasound-assisted extraction is another effective alternative to the common solvent extraction method and very simple to perform. Ultrasound frequencies are here used to cause a mechanical impact that disrupts the plant matrix and induces the formation of cavitation bubbles containing vaporized solvent. At these conditions, it is further easy to extract bioactive compounds. Thus, the extraction yield is highly dependent on the ultrasound frequency and nature of the plant matrix. It should be noted that the production of free radicals might occur in some cases, and then, alter the final bioactive compound content. In tomatoes, the ultrasound-assisted method is specially used to extract lycopene and β-carotene similarly to the previous processes [100, 103].

1.5. *Enzymes: Enzyme assisted extraction (EAE)*

The enzyme assisted extraction (EAE) method also emerges as a novel approach of bioactive compounds extraction. In this process, enzymes are used to disrupt the plant cell walls to release the intracellular content. Since the plant cell walls usually contain polysaccharides, like hemicellulose, cellulose, and pectin, it is necessary to use enzymes that target these subtracts. As an example, β-glucosidase, cellulase, and pectinase are some of the main used enzymes to disrupt the plants´ matrix and depolymerize the polysaccharides, thus allowing the extraction of several compounds of interest. The principle of the EAE method is to catalyze the cell wall disruption in an aqueous solution in mild conditions. Nowadays, this technique is most applied to extract antioxidants from food waste. Just like the techniques mentioned earlier, it is a non-toxic and environment friendly method using water as a solvent, thus being referred as a "biological-refinery". Additionally, other advantages have been highlighted, such as lower energy consumption and solvent use besides short extraction time. The enzymes used for extraction can be added either alone or together. Some preparations of mixed enzymes allow obtaining improved recovery rates. In fact, the proper enzyme combination will determine optimal extraction yields. Preparations composed of pectin- and cellulose-degrading enzymes have shown to be an interesting alternative for lycopene extraction in tomato skin when compared to other enzyme assisted extractions. In tomato matrix, some studies have shown efficient extractions of phenolic compounds and carotenoids when using the EAE method. Accordingly, higher recovery rate of lycopene from tomato waste (peel) or paste has been reported. The enzyme combinations arise as more affordable and with possible industrial-scale applications [100, 104].

1.6. Microwave: Microwave-assisted extraction (MAE)

The microwave-assisted extraction (MAE) is an innovative method that combines the conventional solvent extraction technology with microwaves. The principle of the MAE method is the separation of solutes from plant matrices by inducing diploid rotation and ionic conduction and by transforming microwave energy into heat. When selecting the solvent for this method, it is of great importance to consider the plant matrix, target compounds′ polarity and structure, toxicity, and economical cost. Some of the most used solvents are water, ethanol, hexane, among others, used either alone or combined. To increase the extractions rates, other solvents may be also added, for example, sodium hydroxide or acid chloride. Some of the advantages of this technique are improved extraction rates, short extraction periods, lower solvents requirements, and costs when compared to conventional methods. The MAE can be used for the extraction of different compounds, including polysaccharides, polyphenols, etc. However, it has not been widely used for tomato extraction yet. According to few reports, the extraction of polyphenols and flavonoids from tomato peel waste using the MAE method presented an extraction recovery rate highly influenced by temperature, solvent mixture, and extraction time. To achieve optimal recovery rates, further studies are still necessary. Nevertheless, the MAE method arises as a promising alternative for the extraction of polyphenols in tomato waste, with improved and fast recovery [100, 105].

Table S1 presents an overview of all the techniques for the extraction of bioactive compounds from tomatoes.

Table S1. Summary of advantages, disadvantages and extracted compounds in tomatoes by different methods.

Method	Advantages	Disadvantages	Extracted compounds
Solvent Extraction (SE)	• Low processing costs<br>• Simple performance<br>• High carotenoids recovery rate	• Toxic solvent use<br>• Environmental and health hazard<br>• Decreased added value of extracts	• Lycopene<br>• Anthocyanins<br>• β -carotene
Supercritical Fluid Extraction (SFE)	• Environmentally friendly<br>• Non explosive<br>• Inexpensive (except the equipment)<br>• Increased nutrient retention<br>• Extraction of lipophilic compounds	• Selection of the proper supercritical fluid for optimal extraction	• Lycopene<br>• β-carotene<br>• Phenolic compounds<br>• Tocopherols
Supercritical Water Extraction (SWE)	• Environmentally friendly<br>• Non-toxic<br>• Low costs<br>• Short extraction times	• No studies available yet for the extraction in tomatoes	• Phenolic compounds
Ultrasounds	• Simple to perform<br>• Higher yields of recovery	• Highly dependent on the ultrasound frequency and nature of the plant matrix<br>• Production of free radicals that can alter the bioactive compound content	• Lycopene<br>• β-carotene
Enzyme	• Non-toxic<br>• “Bio-refinery”<br>• Increased extraction rates	• Proper enzyme combinations	• Phenolic compounds<br>• Carotenoids

	• Lower energy consumption<br>• Less solvent consumption<br>• Faster extractions<br>• Affordability<br>• Possible industrial scale application		
Microwave	• Improved extraction rates<br>• Short extraction period<br>• Lower solvents requirements<br>• Lower costs	• Toxicity<br>• Highly influenced by temperature, solvent and duration of the process<br>• Few studies available to this date	• Polysaccharides<br>• Polyphenols

While most of these techniques can be used to successfully extract lipophilic compounds, not all are suited for the extraction of hydrophilic compounds like vitamin C. The solvent extraction method (SE) can be used for the extraction of any compound, as long it is soluble in the solvent used for the extraction. Techniques like Supercritical water extraction (SWE) could also be an interesting alternative for the extraction of hydrophilic compounds. Pressurized liquid extraction (PLE) although not here explored is equally an alternative technique for the extraction of vitamin C from foods. Since there are not studies available studying such extraction in tomato wastes, further research is necessary. Since microwave and ultra-sound assisted extractions also use solvents during their extraction procedures, eventually the extraction of compounds like vitamin C (if the right solvents are applied) would also be a possibility. Yet temperature and the formation of free radicals that interfere with these techniques respectively may result in inefficient extractions [100, 106].

When talking about the extraction of bioactive compounds from food waste there are additional challenges to consider. While the extraction of bioactive compounds from whole foods is already challenging especially due to instability (once removed from its original matrix) when extracting target compounds from food waste the low concentrations that can be extracted are one of the main challenges [100, 107].

Nevertheless, once extracted purity and content should be readily accessed through chromatographic methods as High-Performance Liquid Chromatography (HPLC), spectrophotometric or others according to the bioactive of interest [108-110].

2. Effects of Oral Supplementation and/or Topical Application of Vitamin C, Lycopene, β-Carotene, Palmitic acid, and Zinc

The concept of ingesting bioactive compounds to improve skin outlook and overall health is becoming even more significant. In fact, the human skin is continuously exposed to external aggressors, such as solar UV radiation, pollution, smoke, among others which are related with the formation of reactive oxygen species (ROS), namely free radicals. ROS are highly unstable and when in high levels can be toxic and damage the cell membranes, proteins, and even DNA [111, 112]. While the application of topical formulations to neutralize such compounds appears, at first hand to be enough, some researches show that enriching the skin from within is also important to reduce the impact of those external stressors. Although there are not available oral and/ or topical treatments yet for a huge variety of skin disorders, there is a consensus regarding the importance of maintaining a healthy state including a well-hydrated skin, the right balance of the microbiome film, and the essential skin nutrients in order to retain its barrier integrity and properties [111-114].

Vitamin C is an anti-inflammatory agent due to modulation of the nuclear factor kappa-light-chain-enhancer of activated B cells (NF-κB) transcription factor and a powerful antioxidant able to neutralize the ROS and regenerate vitamin E. It is also essential for different physiological processes such as collagen biosynthesis, protein metabolism, etc. The presence of this vitamin in the human body relies solely on diet and/or oral supplementation to reach the standardized daily values. It is important to note that even when supplementing high doses of vitamin C, only a small fraction is actively bioavailable on the skin. Thus, the association of oral supplementation with topical vitamin C may be a good start point of research to evaluate the real benefits and additive outcomes [111, 112, 115]. Most studies suggested that improved nutritional vitamin C intake is more associated with skin benefits compared to topical formulations (mostly anti-aging and anti-pigmentation creams) [112, 114].

Additionally, it has been suggested that vitamin C can play a major role in the prevention and modulation of other diseases, such as cancers, cardiovascular disorders, age-related macular degeneration, and cataracts, in which oxidative stress plays an important role [115].

β-Carotene is a precursor of vitamin A that exhibits reactive species scavenging properties, and therefore, protection against UV- induced photodamage [111, 116]. When it comes to oral β-carotene supplementation, studies suggest that it can improve the skin outlook and protect it against sunburn reactions [79, 111]. Other health benefits have been associated with β-carotene supplementation combined with other antioxidants (e.g. vitamins C or E). Additionally, diets with a higher content of β- carotene and α-carotene have also been associated with reduced incidence of type 2 diabetes [117]. Nevertheless, it is important to note that a continuous β-carotene supplementation, specifically at high cumulative level (above physiological levels), might be associated with negative side effects. In particular, some concerns were raised in populations with respiratory conditions [79].

Other carotenoids from tomatoes, such as lycopene, have also been shown to improve the skin´s tolerance to UV radiation. Studies demonstrated that the consumption of tomato-based products, that nourished the body with around 16 mg of lycopene, reduced the sun- induced erythema, demonstrating that lycopene exhibits photoprotective activity when ingested [79, 111]. Other reports showed that lycopene supplementation could improve the endothelial health in patients with cardiovascular disease [118]. Additionally, one study concluded that oral lycopene supplementation in middle-age individuals allowed the lycopene skin accumulation up to levels similar to younger individuals [119]. Lycopene has demonstrated to own photoprotective properties being a powerful quencher of ROS whether ingested or topically applied. As it has a lipophilic profile and reduced molecular size, enhanced skin absorption is observed when it is incorporated in topical formulations. Therefore, researchers are investigating the best topical systems that allow lycopene to retain its properties and delay its degradation taking into account the low chemical stability of this molecule [107].

Palmitic acid is the most abundant saturated fatty acid on the skin and possesses a crucial role in epidermal morphogenesis. The topical use of palmitic acid is commonly related to skin lipids replacement and skin repair whether its supplementation is used to treat and/or prevent certain skin disorders. However, high amounts of palmitic acid can lead to the inhibition of linoleic acid and thus to undesired effects [120, 121].

Zinc is an essential nutrient for the proper function of the human metabolism and immune system besides playing an important role in healthy skin. Zinc is highly abundant in the skin, and it is especially important for wound healing. This bioactive has shown to modulate the expression of inflammatory factors and to exhibit antioxidant properties. Some researchers have suggested that diseases with skin manifestations can be caused by mutations or dysregulations in zinc transporters that could lead to its deficiency. Zinc deficiency affects mostly the epidermis, and gastrointestinal, skeletal, nervous, reproductive, and immune systems. Additionally, if it occurs during the growth period it can lead

to growth failure. Oral zinc supplementation is generally safe and beneficial and necessary for those with lower levels, but when it comes to skin manifestations, topical treatments may be enough. Formulations containing zinc have been used over years as active ingredients in antidandruff products, as soothing agents, and even in sunscreens. More recently, other applications have emerged as the treatment of pigmentary disorders (melasma), inflammatory dermatoses (rosacea and acne vulgaris), and even neoplasia [122-124].

At last, it is important to consider that while oral supplementation appears to be more efficient to lead to desired outcomes, daily values should be respected and not exceeded. Lipophilic molecules as β-carotene are not directly excreted through urine like hydrophilic compounds (Vitamin C). Some of these molecules have to first undergo metabolization processes before being excreted. Therefore, they present lower daily values to avoid accumulation and potential side-effects.

Additionally, while antioxidants have many described benefits they can work as pro-oxidants instead in certain situations. The pro-oxidant effect may occur when the antioxidants are in high concentrations or depending on matrix's nature and redox potential. Vitamin C for example although being a powerful antioxidant can work as a pro-oxidant when administered in concentrations around or higher than 1000 mg/kg of bodyweight. Vitamin C can also act as pro-oxidant in the presence of minerals such as iron and copper when participating in redox reactions. The same happens to α-tocopherol that acts as a pro-oxidant in high concentrations, to the point of working as a free radical instead. If not enough vitamin C is available to reduce these tocopherol molecules, they remain in their reactive state and lead to undesired side effects [125, 126].

3. Approaching the tomato food producing industry to the cosmetic and pharmaceutical industry by using waste?

Taking into consideration what was previously discussed, tomato by-products present themselves as great raw materials to explore innovative applications towards cosmetics and nutraceuticals besides other health products.

Over the past years, the trend is to take care of the skin inside-out. Although new delivery systems are being developed, with the ability to target and deliver the active ingredients to the exact location, additional studies recognize the importance of nutrition (including diet and oral supplements) for guarantee the skin health and overall well-being [111, 114, 127].

Most tomato by-products contain active ingredients with photoprotective, antioxidant and anti-inflammatory activities, thus the further application of these compounds either in topical or oral formulations could be an interesting alternative for tomato producing industries instead of disposing these compounds as industrial waste. Incorporating lycopene, β-carotene, vitamin C, palmitic acid, and zinc sustainably obtained from tomato by-products, into supplements and topical formulations would allow building a bridge between the food industry and pharmaceutical and cosmetic industries. This bridge would allow us to further approach the concept of using natural whole foods to treat and prevent diseases from the inside-out point of view. Notwithstanding, further, and rigorous studies are still necessary to become more consensual. Anyway, it becomes clear that it is no longer possible to target disorders at only one pathway. Lastly to enhance public acceptability, color, smell, and texture of these formulations, especially in cosmetics should be improved [111, 113, 114].
